# Silver and Antibiotic, New Facts to an Old Story

**DOI:** 10.3390/antibiotics7030079

**Published:** 2018-08-22

**Authors:** Frédéric Barras, Laurent Aussel, Benjamin Ezraty

**Affiliations:** 1 Laboratoire de Chimie Bactérienne (LCB), Institut de Microbiologie de la Méditerranée (IMM), Aix Marseille Université, Centre National de la Recherche Scientifique (CNRS), 13009 Marseille, France; aussel@imm.cnrs.fr; 2 Département de Microbiologie, Institut Pasteur, 75015 Paris, France

**Keywords:** silver, antibiotics, adjuvant, combinatorial, metal, ROS

## Abstract

The therapeutic arsenal against bacterial infections is rapidly shrinking, as drug resistance spreads and pharmaceutical industry are struggling to produce new antibiotics. In this review we cover the efficacy of silver as an antibacterial agent. In particular we recall experimental evidences pointing to the multiple targets of silver, including DNA, proteins and small molecules, and we review the arguments for and against the hypothesis that silver acts by enhancing oxidative stress. We also review the recent use of silver as an adjuvant for antibiotics. Specifically, we discuss the state of our current understanding on the potentiating action of silver ions on aminoglycoside antibiotics.

## 1. Introduction

The antibacterial effect of silver ions (Ag^+^) has been known for centuries as ancient Greek used silver for stomach pains or wound healing. According to Mijnendonckx et al. [1], “silver was perhaps the most important antimicrobial compound before the introduction of antibiotics”. Currently, it is used on surfaces in hospitals to reduce nosocomial disease. It is also widely used in water cleaning systems such as hospital hot water circuits, swimming pool and potable water delivery systems. And as Simon Silver repeatedly recalled it, silver can even be found within Japanese Jintan pills meant “to cure from nausea, vomiting, hangover, bad breath and sunstroke among others” [1]. More recently, a series of initiatives aimed at fighting multidrug resistant bacteria elected combinatorial strategies as a way of potentiating drug efficiency [2]. Likewise, silver ions were identified as a highly efficient potent of antibiotics of different classes [3,4]. Last, silver nanoparticles (AgNP) rank currently among the most widely commercialised nanomaterial used in medical, bactericidal and electrical products [5]. Despite this old and broad use, the mechanism underlying antimicrobial activity of silver ions is not fully understood. 

In this Review, we will first describe the multiple cases of silver ions being used as biocides. We will then give a broad overview of the many situations wherein combining silver and antibiotics yielded to enhanced antibacterial efficiency. Last, the molecular mechanism allowing silver to potentiate aminoglycoside toxicity will be discussed. Strategies based upon silver nanostructures, as well as their synthesis, toxicity and efficiency, will not be covered in the present review and interested readers can find examples of such studies in References [6,7,8].

## 2. Molecular Basis of Silver Toxicity toward Microbes

**Figure 1:**
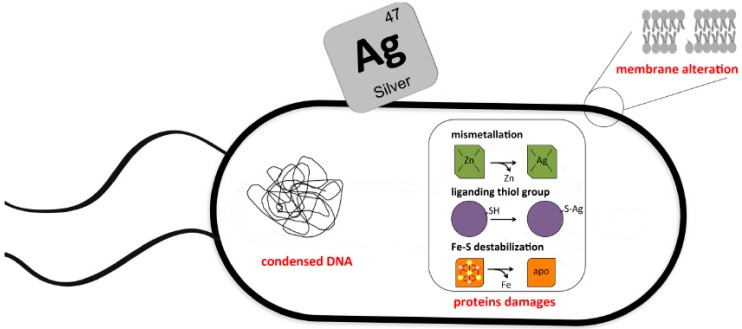
Pleiotropic molecular basis of antimicrobial effects of silver. Silver targets different macromolecules in bacteria. Here are depicted modifications observed in silver-treated bacteria such as DNA condensation, membrane alteration and protein damages. In this latter case, several situations were reported wherein silver ions interacted with thiol group, destabilised Fe-S clusters or substituted to metals in metalloproteins.

Silver antibacterial activity has been studied for a long period of time [9]. Silver ions were proposed to target macromolecules and their associated alteration was predicted to be the cause of silver mediated toxicity (Figure 1). Yet, although some consistent trend emerged from this bulk of studies, several discrepancies remained. 

### 2.1. Silver Ions Target DNA

Silver ions are strong nucleic acids binders and form several complexes with DNA or RNA. They interact preferentially with bases rather than the negatively charged backbone of DNA. Thermodynamic experiments showed that silver ions formed homo-base pairs with a higher affinity with guanine, which could potentially lead to pyrimidine dimerization. At a high concentration, silver ions were observed to interact with adenine [10]. Microscopy analysis of silver treated bacteria showed a dense electron-light region assigned as condensed DNA in the centre of the cells. While all of these in vitro observations support the hypothesis that silver could lead to DNA modification prone to mutation or replication inhibition, actual contribution of DNA-silver adducts formation to silver antimicrobial toxicity remains to be assessed in vivo. 

### 2.2. Silver Ions Target Proteins

A silver target on which everybody agrees is the sulfhydryl group, which results in the formation of S-silver bond [11]. Sulfhydryl groups belong to lateral chains of Cys residues. Cys residue frequently served as ligand for metal and/or cofactors in metalloproteins, including those forming respiratory chains. Accordingly, silver ions were found to alter respiration of *E. coli* [9,12] and it was thought that proton motive force (PMF) collapse due to respiration inhibition constituted the basis of silver toxicity [13]. However, subsequent work revealed that silver had additional targets besides respiration [14]. For instance, in *Vibrio cholerae*, proton leakage, which could be a consequence of PMF collapse, was observed even in the absence of the NADH-ubiquinone oxidoreductase [13]. This suggested that silver had multiple protein targets in the membrane. Recently, Xu and Imlay investigated the toxicity of different soft metals in *E. coli* and identified Fe-S cluster containing proteins as primary targets of silver [15]. Importantly, NADH dehydrogenase I activity, a main component of the aerobic respiratory electron transfer chain, was untouched by silver treatment. Instead dehydratases like fumarase A appeared as preferred targets. The 4Fe4S cluster from fumarase was degraded to 3Fe4S cluster that could be reactivated by exogenous Fe^2+^ under reducing conditions. The reason for the apparent specificity of silver ions for Fe-S cluster from dehydratases likely stems from the exposed nature of their solvent and the lability of the catalytic Fe atom. 

Other candidate targets include thiol containing cytoplasmic proteins. For instance, OxyR, the H_2_O_2_-sensing transcriptional activator, was reported to be inactivated in silver-exposed *E. coli* strains [3]. The authors argued that silver antagonises disulphide bond formation within OxyR monomer, which is required for activating transcription. 

Last, one should keep in mind that the high thiophilicity of silver ions could allow them to substitute for any SH-liganded metal. For instance, it is conceivable that silver acts upon Fe-S cluster by substituting for labile Fe atom, which would apply to the dehydratase situation depicted above. Alternatively, silver could substitute for zinc ions in, for instance, zinc-finger proteins. Overall, these substitutions could lead to massive protein mis-metallation, loss of function and associated defects. It is noteworthy that cytosolic dense granules were observed in silver treated *E. coli* cells; such granules were interpreted as being constituted of misfolded protein aggregates [3].

### 2.3. Silver Mediated Membrane Alteration

Transmission electronic microscopy (TEM) observation of silver treated *E. coli* revealed morphological and structural changes of the cell envelope. Moreover, use of propidium iodine showed an enhanced permeability of the cell envelope [3]. In a separate study, TEM revealed an enlargement of the periplasmic space in *E. coli*, suggesting the shrinking of the inner membrane and its detachment from the cell wall. Interestingly a gram-positive bacterium, *Staphylococcus aureus*, which exhibits a thicker cell envelope underwent similar morphological changes than *E. coli*, albeit to a lesser extent, suggesting a stronger resistance to silver ions [16]. 

### 2.4. Are Silver Ions Producing ROS?

There is much debate on whether silver, which is not a redox active metal, induces ROS formation, and if it is the case, how this happens. To determine whether silver ions induce ROS, Park et al. used a *soxS-lacZ* reporter strain. After exposure to silver nitrate, induction of *soxS* was observed. As *soxS* expression being under the control of SoxR, it was deduced that superoxide radicals had been produced by the presence of silver ions. However, no *soxR* control mutant was tested and the actual signal SoxR is responding to remains a matter of debate. In particular, it has been proposed that SoxR senses the ratio NAD(P)H/NAD(P) [17]. Were silver ions to impair respiration, this ratio would be modified and SoxR activated without the need for superoxide production. Importantly OxyR activation was not observed, supporting the notion that no H_2_O_2_ accumulated in the presence of silver ions. Using 3′-p-hydroxyphenyl fluorescein (HPF), a dye, Morones et al. observed hydroxyl radicals production in silver treated *E. coli* cells [3]. Surprisingly, overproduction of superoxide dismutase (SOD), predicted to enhance hydroxyl radical production via the Fenton reaction, was found to reduce HPF-estimated hydroxyl radical. Moreover, detection of hydroxyl radicals by Morones et al. [3] somehow did not fit with the lack of H_2_O_2_ enhanced production and lack of OxyR induction reported by Park et al. [18]. If ROS were instrumental in conveying silver toxicity, a prediction is that anaerobically grown cultures should be less sensitive to silver ions. This issue was investigated in several studies but unfortunately conflicting observations were reported and it is so far impossible to draw a firm conclusion from the literature (see [1]). Last, a very recent transcriptomic analysis of *E. coli* exposed to silver ions failed to identify anti-ROS defence genes induction, while dysregulation of silver transport and detoxification (*copA*, *cueO*, *mgtA*, *nhaR*), stress response genes (*dnaK*, *dnaJ*, *pspA*, oxidoreductase genes), methionine biosynthesis (*metA, metR*), membrane homeostasis (*fadL*), and cell wall integrity (*lpxA*, *arnA*, *ycfS*, *ycbB*) were identified [19]. Hence, experimental evidences for silver ions to induce ROS production remain scant and open to discussion.

**Figure 2:**
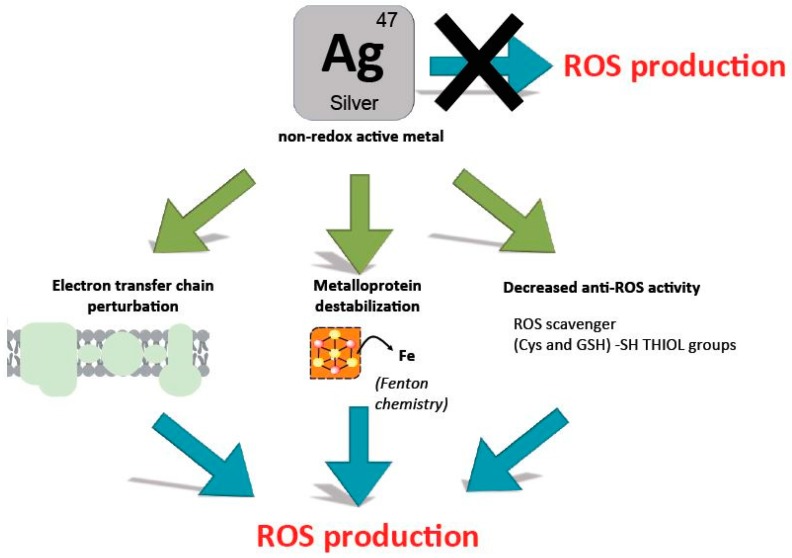
Searching for the causal link between silver ions and ROS production. Silver is a non-redox active metal that cannot directly produce ROS. Some experimental evidences however pointed to the enhanced production of ROS in the presence of silver ions. Depicted here are possible indirect ways silver ions could participate to ROS production: Perturbation of respiratory electron transfer chain, Fenton chemistry following destabilization of Fe-S clusters, or displacement of iron, inhibition of anti-ROS defences by thiol-silver bond formation.

On the other hand, if one admits that silver ions are perturbing iron homeostasis as well as destabilizing Fe-S clusters, it seems quite likely that eventually this will indirectly lead to ROS production (Figure 2). Indeed, because Fe-S proteins are central to respiration, this latter is expected to be perturbed and this could provoke electron leakage and associated ROS production. Also, destabilization of Fe-S clusters is expected to release free iron, which should fuel in the Fenton reaction. Last, silver ions by binding to thiols will preclude endogenous anti-ROS defences such as free cysteine and glutathione, two compounds with ROS-scavenging properties. 

Hence it seems indeed a safe prediction that silver ions will favour ROS production, yet the causal chain linking silver, a non-redox soft metal, and ROS production remains to be established and described in precise molecular terms. 

## 3. Silver Enhances Antibacterial Activity of Antibiotics

In 2007, Morones et al. investigated the capacity of silver ions to synergise antibiotics [3]. They reported that silver potentiates bactericidal antibiotics both in laboratory growth conditions and animal models. The three major classes of bactericidal antibiotics in *E. coli* were tested, i.e., ß-lactams (ampicillin), which target cell-wall synthesis, quinolones (ofloxacin), which target DNA replication and repair, aminoglycosides (gentamicin) that are ribosome binders known to cause protein mistranslation. All of these drugs were tested at a concentration close or inferior to the MIC values, and in the presence of sublethal concentrations of silver. In all of these cases, a significantly enhanced antimicrobial activity was observed. A more precise analysis revealed that the highest synergistic effect was found when combining gentamicin and silver as viability dropped 2 logs. In the case of ampicilin and ofloxacin, presence of silver decreased viability 1 log at the maximum. After showing that mice tolerated the silver concentration used (3–6 mg/kg), the authors reported that silver potentiated both the gentamicin activity in a urinary tract infection mouse model, and the vancomycin activity in a mouse peritonitis infection mouse model. 

The potentiating activity of silver on antibiotic toxicity in *E. coli* K12 was further investigated by Herisse et al. [4]. An extended set of bactericidal and bacteriostatic antibiotics including tetracycline and chloramphenicol were tested [4]. According to changes in MIC values, silver was found to be most potent with aminoglycosides (gentamicin, kanamycin, tobramycin, streptomycin) as MIC value decreased by more than 10-fold. A reduction in the MIC value of 2-fold was noted with spectinomycin, a bacteriostatic antibiotic related to aminoglycoside and also with tetracycline. Moreover, they reported a slight potentiating effect (less than 20%) when silver was used in conjunction with quinolone (nalidixic acid and norfloxacin) or with chloramphenicol [4]. 

Another study showed that silver enhances the toxicity of the selenazol drug ebselen, a competitive inhibitor of bacterial thioredoxin reductase activity against clinically multidrug-resistant Gram-negative bacteria (*Klebsiella pneumoniae*, *Acinetobacter baumannii*, *Pseudomnas aeruginosa*, *Enterobacter cloacae*, *Escherichia coli*) [20]. Potentiating effects were observed both in laboratory growth conditions and mice peritonitis model (6 mg/kg). Similarly, Wan and collaborators in a study on AgNP showed that ionic nitrate silver acts synergistically with polymixin B and rifampicin to combat carbapenem-resistant *A. baumannii* obtained from clinical patients. Interestingly, AgNP and AgNO_3_ showed the same potentiating effect with both antibiotics, but cytotoxicity of AgNP was lower than that of AgNO_3_ [21]. Silver was also reported to potentiate polymixin B and a series of antimicrobial peptides to combat gram-negative bacteria [22].

Many antibiotics that are effective against planktonic cells turned out to be ineffective against biofilms. Combination of silver with tobramycin combated biofilm of *E. coli* and *Pseudomonas aeruginosa* as a 3-fold enhancement of antimicrobial efficiency was observed [23]. A similar potentiating effect of silver (6 mg/kg) with gentamicin was noted in combating biofilm formed on a catheter located into a mouse model [3].

Last, silver made antibiotics effective against resistant bacteria. Indeed, silver was able to sensitise *E. coli* to the Gram-positive-specific antibiotic vancomycin and the highly tolerant anaerobic pathogen *Clostridium difficile* became sensitive to aminoglycoside [3,4]. Moreover, silver could restore antibiotic susceptibility to a tetracycline resistant *E. coli* mutant [3].

We wish to underline that the use of silver as an adjuvant might also be of interest to treat persister cells, a subpopulation of isogenic bacteria that become highly tolerant to antibiotics [3]. All these data are grouped in Table 1.

## 4. Molecular Mechanism in the Aminoglycoside/Silver Synergy

**Figure 3:**
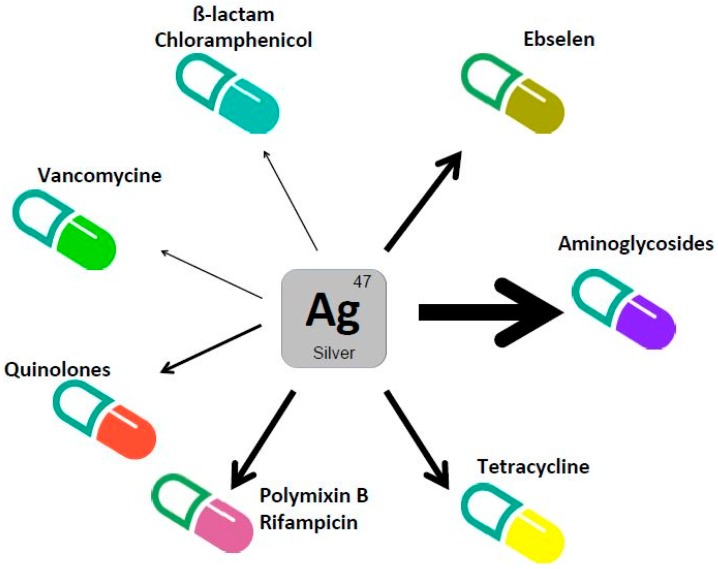
Silver potentiates antibiotics toxicity. The capacity of silver ions to enhance the toxicity of antibiotics from different family is represented. The size of the arrows line reflects the extent of the synergistic effect.

Of all antibiotics tested, aminoglycosides (gentamicin, tobramycin, kanamycin, streptomycin) benefited the most from silver ions as adjuvants (Figure 3). The molecular basis of the synergistic effect between silver and aminoglycoside has been investigated in two separated studies, which we discuss below [3,4]. However, we shall first recall how aminoglycosides are predicted to kill bacteria, and in particular how they are uptaken by *E. coli*. 

Aminoglycosides, first discovered in the 1940s, are the antibiotics most commonly used worldwide, due to their high efficacy and low cost [24]. Aminoglycosides are a group of bactericidal antibiotics that target the 30S ribosomal subunit and induce amino acid mis-incorporation. Aminoglycoside need to be transported through the cytoplasmic membrane to reach their target. These transport systems are energised via proton motive force (PMF)-dependent pathways [25]. Moreover a so-called feed-forward loop model postulates the occurrence of a two-steps process: Aminoglycosides would cross quite inefficiently the cytoplasmic membrane prior to hit membrane-bound ribosome (EDP-I), resulting in aborted translated products, which would go into the membrane due to their hydrophobic characters, and destabilise further the membrane, allowing for enhanced entry of aminoglycoside (EDP-II) [26]. 

**Figure 4:**
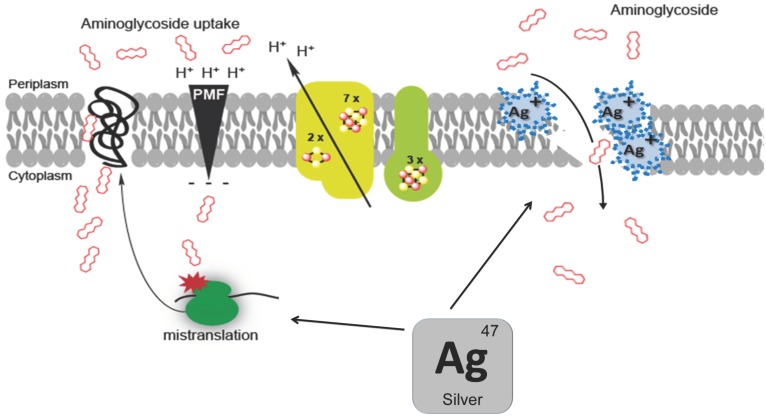
A molecular mechanism model for aminoglycoside and silver synergy. Silver enhances aminoglycoside toxicity by enhancing their uptake. Silver could destabilise the membrane either directly by altering intrinsic membrane proteins or indirectly by acting on ribosomes, which would produce misfolded aborted polypeptides that would eventually go to the inner membrane. Increased permeability of membrane would provoke massive aminoglycoside uptake.

Herisse et al. showed that silver enhances aminoglycoside toxicity by acting independently of PMF as it by-passes the EDP-I PMF-dependent step of the aminoglycoside entry process. Silver by-passed the antagonist effect of the PMF dissipating action of the carbonyl cyanide-m-chlorophenylhydrazone (CCCP), an uncoupler H^+^ ionophore [4]. Moreover, silver restored aminoglycoside uptake by strains exhibiting a reduced PMF level such as mutants lacking complex I and II (∆*nuo* ∆*sdh*) or Fe-S cluster biosynthesis (∆*iscUA*) [4]. In contrast, the silver-potentiating effect of aminoglycoside toxicity remained dependent on translation, the EDP-II proteins translation-dependent step [4]. Indeed, adding chloramphenicol, a bacteriostatic antibiotic inhibiting translation, prevented silver from potentiating aminoglycoside toxicity. It was proposed that silver destabilises the membrane in a protein translation-dependent pathway, allowing aminoglycoside to get access to the cytosol more efficiently. This implied that membrane disturbance induced by silver is not sufficient for massive aminoglycoside uptake and needs additional contribution from mis-localised aborted polypeptides. By acting directly on ribosomes, silver could release aborted translated products that would eventually go to the membrane and cause an EDP-II like step (Figure 4). This agrees with a proposal by Morones et al. [3] who envisioned that silver produced misfolded proteins would be directed towards the inner membrane and destabilised it. An argument supporting this view was that enhanced silver resistance of a *secG* mutant impaired in protein translocation [3]. Hence, irrespective of the origin and cause of increased level of misfolded proteins, both studies pointed out to an enhanced permeability of the cell envelope. This is consistent with morphological and structural changes observed by TEM studies of silver treated cells (see above). 

In contrast, the contribution of ROS to silver toxicity was more controversial. Morones et al. postulated that silver ions enhance gentamicin toxicity via the capacity of silver to produce ROS [3]. However the enhanced production of ROS in the presence of the combination (silver+gentamicin) was not tested. Moreover, the actual production of ROS following silver addition is highly debatable as summarised above. Last, Herisse et al. directly addressed the question of the contribution of ROS to the silver potentiating effect and collected only negative evidences: (i) silver potentiated gentamicin toxicity even in anaerobic conditions; (ii) mutants altered in anti-ROS activities like the strains lacking superoxide dismutases (∆*sodA* ∆*sodB*) or the H_2_O_2_-stress responding master regulator (∆*oxyR*) exhibited similar sensitivity to silver potentiating effect as the wild type *E. coli* [4].

## 5. Conclusions

In this review we listed numerous cases in which silver ions were reported to exhibit efficient antibacterial activity. We also reviewed the emerging trend of using silver ions as adjuvants for potentiating antibiotic toxicity. It is compelling that after so many years, the actual reason silver kills bacteria is still eluding us. In fact, it is likely that silver ions act upon multiple different targets, from macromolecules to free amino-acid like cysteine or small molecule such as glutathion, and therefore renders it difficult, if not impossible, to trace the actual cause of death of a silver treated bacterium. Nevertheless, some pressing issues remain: Is silver destabilising protein components of respiratory chains? Does silver have any deleterious (mutagenic?) effect on genome integrity? What is the actual structural state of a silver-destabilised membrane? What is the link, if any, between silver and ROS production? 

There is little doubt that new efforts should be dedicated towards the understanding of the action of silver such that this very ancient antibacterial metal can be further exploited within the context of the multiple antibiotic resistance crisis. Interest for such a potential path is reinforced by the fact that pharmacological, toxicological and pharmacokinetic modelling studies indicated that human health risks associated with silver exposure were low [27,28]. From a broader perspective, recently, we advocated the need to take into account iron in its influence on antibiotic sensitivity [29]. It is known that most metals can have antibacterial activities at high concentration, such as bismuth, cobalt, copper and cadmium, to cite a few [15,30,31,32]. Aiming at characterising and further exploiting their biocide activity might be a rewarding goal.

## Figures and Tables

**Table 1:** Antibacterial activity of silver ions in combination with antibiotics.

Antibiotics		Organism	Culture Condition	Effects	References
**ß-lactams**	Ampicillin	*E. coli*	Laboratory medium	10-fold increase in antimicrobial activity	[3]
**Quinolones**	Ofloxacine, Nalidixic Acid, Norfloxacin	*E. coli*	Laboratory medium	10-fold increase in antimicrobial activity. MIC value decreased 10–25%	[3,4]
**Aminoglycosides**	Gentamicin	*E. coli*	Laboratory medium. Animal models	100-fold increase in antimicrobial activity. MIC value decreased more than 10-fold	[3,4]
		*C. difficile*	Laboratory medium	MIC value decreased 4-fold	[4]
	Tobramycin	*E. coli.*, *P. aeruginosa*	Laboratory medium	MIC value decreased 10-fold (*E. coli*). 3-fold increase in antimicrobial activity (*P. aeruginosa*)	[4,23]
	Kanamycin Streptomycin	*E. coli*	Laboratory medium	MIC value decreased more than 10-fold	[4]
**Spectinomycin**		*E. coli*	Laboratory medium	MIC value decreased 2-fold	[4]
**Vancomycin**		*E. coli*	Laboratory medium. Animal models	10-fold increase in antimicrobial activity	[3]
**Chloramphenicol**		*E. coli*	Laboratory medium	MIC value decreased 1.5-fold	[4]
**Ebselen**		*K. pneumoniae*, *A. baumanni*, *P. aeruginosa*, *E. cloacae*, *E. coli*	Laboratory medium. Animal models	10-fold increase in MIC value	[20]
**Polymixin B**		*E. coli*	Laboratory medium	MIC value decreased 5- to 10-fold	[21,22]
**Rifampicin**		*A. baumannii*	Laboratory medium	MIC value decreased 5-10 fold	[21]
**Tetracycline**		*E. coli* (Tet^R^)	Laboratory medium	MIC value decreased 2-fold	[3]
